# An Update on Molecular Tools for Genetic Engineering of Actinomycetes—The Source of Important Antibiotics and Other Valuable Compounds

**DOI:** 10.3390/antibiotics9080494

**Published:** 2020-08-08

**Authors:** Lena Mitousis, Yvonne Thoma, Ewa M. Musiol-Kroll

**Affiliations:** Interfaculty Institute for Microbiology and Infection Medicine Tübingen (IMIT), Microbiology/Biotechnology, University of Tübingen, Auf der Morgenstelle 28, 72076 Tübingen, Germany; lena.mitousis@student.uni-tuebingen.de (L.M.); yvonne.thoma@student.uni-tuebingen.de (Y.T.)

**Keywords:** actinomycetes, natural products, synthetic biology, genetic engineering, tools, improvement

## Abstract

The first antibiotic-producing actinomycete (*Streptomyces antibioticus*) was described by Waksman and Woodruff in 1940. This discovery initiated the “actinomycetes era”, in which several species were identified and demonstrated to be a great source of bioactive compounds. However, the remarkable group of microorganisms and their potential for the production of bioactive agents were only partially exploited. This is caused by the fact that the growth of many actinomycetes cannot be reproduced on artificial media at laboratory conditions. In addition, sequencing, genome mining and bioactivity screening disclosed that numerous biosynthetic gene clusters (BGCs), encoded in actinomycetes genomes are not expressed and thus, the respective potential products remain uncharacterized. Therefore, a lot of effort was put into the development of technologies that facilitate the access to actinomycetes genomes and activation of their biosynthetic pathways. In this review, we mainly focus on molecular tools and methods for genetic engineering of actinomycetes that have emerged in the field in the past five years (2015–2020). In addition, we highlight examples of successful application of the recently developed technologies in genetic engineering of actinomycetes for activation and/or improvement of the biosynthesis of secondary metabolites.

## 1. Introduction

Actinomycetes are Gram-positive, mostly aerobic, filamentous bacteria of the phylum *Actinobacteria* [1,2,3,4]. They were initially regarded as organisms, which share morphological characteristics with both bacteria and fungi [5,6] and thus, they were considered as transitional forms between the two groups. This is reflected in the name “Actinomycetes” that was derived from Greek “atkis” or “aktin” (means ray) and “mykes” (means fungus) [7,8]. Actinomycetes have adapted to different ecological niches: terrestrial [9,10,11,12,13,14,15,16,17,18], aquatic [19,20,21,22,23,24,25] and artificial (“manmade”) [26,27]. In many cases, their biological function is indispensable. Some species are involved in decomposing complex mixtures of polymers in dead plants, animals and fungal materials contributing to humus formation and the recycling of biomaterials [28,29]. Actinomycetes can also provide a nitrogen-fixing function in symbiotic associations with plants [30,31] or take part in other complex, ecologically relevant interactions [32,33,34]. The most known example includes interactions with insects, where the antibiotics produced by actinomycetes protect the associated organism (e.g., termites, ants, beetles, wasps or they larvae) against detrimental microorganisms [35,36,37,38,39,40].

However, many researchers have drawn attention to actinomycetes mainly because of their biosynthetic potential for the production of secondary metabolites [41,42,43]. The discovery of the first actinomycete-derived antibiotic (actinomycin) [44] boosted the isolation of actinomycetes from diverse sources and the traditional screening involving the cultivation, extraction of metabolites and disk diffusion-based activity assays and/or chromatographic analysis [43,45,46,47]. Since the “Golden Age” of antibiotic discovery (1940s–1960s), several antimicrobial and other valuable compounds were discovered from actinomycetes. Many of these molecules have been developed to commercial products, including agrochemicals and pharmaceuticals [14,47,48,49,50,51]. Thus, actinomycetes are one of the most prolific and important sources of bioactive compounds. Since the whole genome sequencing of the first actinomycetes, which was the genome of *Streptomyces coelicolor* A3 [52], hundreds actinomycetes genomes were sequenced and annotated. These data revealed that the genomes encode several biosynthetic gene clusters (BGCs) for the production of diverse secondary metabolites. However, only a few of the BGCs are expressed at standard laboratory conditions and, therefore, the biosynthetic potential of many actinomycetes remains unexploited [53,54,55,56,57,58,59,60]. Numerous strategies were developed to induce the expression of the BGCs and access the corresponding products. While general concepts and methods of natural product discovery, including new isolation and strain cultivation methods, (meta)genomics-based approaches as well as modern metabolomics-inspired technologies were highlighted in other reviews [43,45,46,61,62,63,64,65,66,67,68], this review provides the reader with an overview on recent advances in molecular tools for the genetic engineering of actinomycetes.

## 2. Genetic Engineering of Actinomycetes

**Figure 1:**
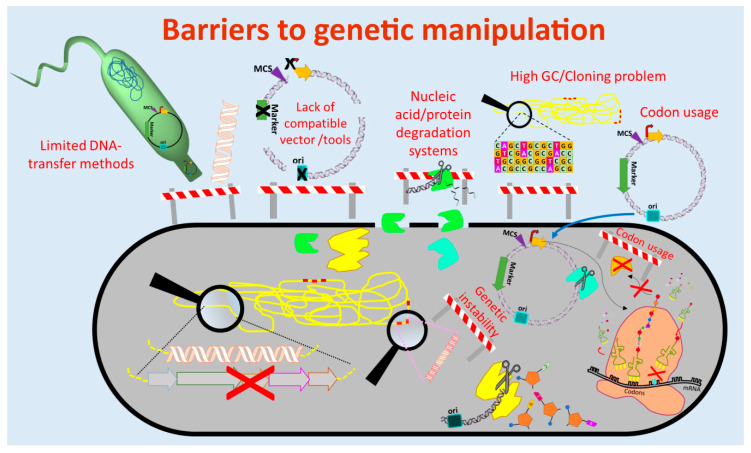
Barriers to genetic manipulation of actinomycetes. Obstacles hindering the genetic manipulation and engineering of actinomycetes include the lack of strain-compatible tools, cloning methods for high guanine-cytosine content (GC-content) DNA sequences as well as transfer methods for introduction of the genetic constructs into the host. Furthermore, nucleic acid degradation systems present in actinomycetes and differences in codon usage may result in fragmentation of the genetic constructs and production of non-functional proteins, respectively. (Single elements of the figure (e.g., ribosome) were re-used from a previous publication [74] with permission from the Royal Society of Chemistry.)

**Figure 2:**
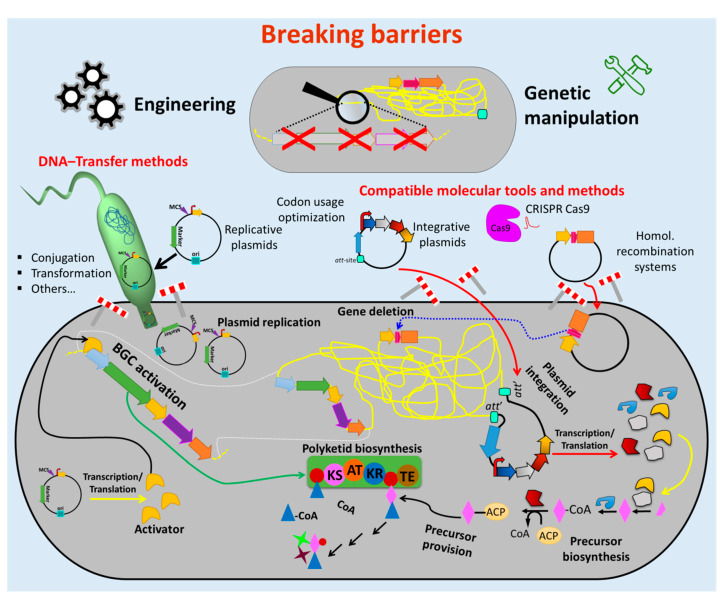
Breaking down the barriers to genetic manipulation and engineering of actinomycetes. Numerous methods and tools were developed to facilitate the genetic manipulation of actinomycetes. Suitable vectors and other vehicles (e.g., integrative and replicative systems) as well as fast and easy cloning strategies were developed to assemble genetic constructs, which are introduced into the host by using different transfer methods (e.g., conjugation and protoplast transformation). Protocols for avoiding DNA-degradation (e.g., treatment of the DNA with NaOH) and solutions for ensuring correct transcription/translation (e.g., codon usage optimization) were established. These innovations led to the generation of many gene deletions and (over)expression mutants (e.g., deletion of competitive biosynthetic pathways, overexpression of activators for activation of silent biosynthetic gene cluster (BGC)). This enormous progress facilitated the metabolic engineering of actinomycetes chassis for manufacturing bioactive natural products, including antibiotics. (Single elements of the figure (e.g., schematic representation of plasmid backbone) were re-used from a previous publication [74] with permission from the Royal Society of Chemistry.).

The currently used genetic engineering strategies for activation of BGC-expression and production of the respective compounds in actinomycetes include the expression of multiple copies of the whole BGC or factors that are limiting the production, expression of activator genes, deletion of genes encoding repressors of the BGC, refactoring of the BGC by substitution or modification of native regulatory elements (e.g., promoters) and/or expression of the BGC in optimized (e.g., genome-minimized, precursor-optimized), native or heterologous hosts [69,70,71,72,73] (Figure 1 and Figure 2). These in vivo engineering approaches require genetic manipulation that depends on molecular tools for assembling of genetic constructs and methods for their introduction into the host. In the past four decades, several barriers were identified that retarded the process of natural product discovery from actinomycetes. Lack of compatible molecular tools, limited cloning and DNA transfer methods, DNA degradation, genetic instability, high guanine-cytosine content (GC-content) of the genomes, and different codon usage of the newly introduced foreign DNA are barriers that often prevent the genetic manipulation of actinomycetes (Figure 1).

Therefore, enormous efforts have been undertaken to develop tools and methods to overcome these obstacles (Figure 2). Advances in sequencing technics [75] and bioinformatics tools for genome mining [63] facilitate cost-effective sequencing and a fast identification of BGCs and other potential targets for genetic engineering. Molecular biology enzymes [76,77,78], fast cloning strategies and synthetic biology which involves vectors and genetic parts (e.g., attachment sites, replicons, selection markers, promoters, terminators) [79,80,81,82,83,84] were optimized for introduction and maintenance of additional or new genetic material in actinomycetes strains. Methods for transfer of the generated construct into the cell (e.g., protoplast transformation, conjugation) were established [74,85].

During the past five years (2015–2020) a variety of technologies and protocols for engineering of actinomycetes genomes have been established. Therefore, we believe that a summary of the recent developments will be a valuable reference for the community focusing on actinomycetes and their natural products. To this end, we present an update on the molecular tools and methods for genetic engineering of actinomycetes. In the first part, details on new cloning strategies were described (Section 2.1). The second part contains an overview on recent advances in “genetic bricks” and molecular tools for genetic manipulation of actinomycetes (Section 2.2). As there is tremendous progress in the development of cloning strategies (Section 2.1) and CRISPR/Cas (clustered regularly interspaced short palindromic repeats/CRISPR associated)-based tools (Section 2.2.4), these two sections have a “lexicon-like” structure. This should ensure an easy and time-saving search for the diverse tools and their explanation.

### 2.1. Assembly Strategies for Generation of Constructs for Genetic Engineering of Actinomycetes

DNA assembly and cloning strategies are indispensable techniques in the field of synthetic biology. Cloning of actinomycete sequences is especially challenging because of the high GC content genomes [48,86] and highly conserved and repetitive sequences (e.g., polyketide synthase (PKS) or nonribosomal peptide synthetase (NRPS) BGCs). Often, the BGC of interest spans more than 100 kb [87,88] and therefore efficient, fast and easy cloning tools are required. In the last five years (2015–2020), some new methods and updates of established protocols for cloning and assembling of genetic constructs were reported. Their successful application was demonstrated in bacterial strains including actinomycetes (Table 1).

#### 2.1.1. iCatch

The iCatch [89] is an upgrade of the 2014 proposed iBrick principle [90] based on the BioBricks concept [91]. This tool was developed to facilitate “catching” of large regions (e.g., BGCs from actinomycetes). The BioBricks concept is based on the creation of DNA pieces flanked by XbaI and SpeI restriction sites, resulting in compatible cohesive ends after restriction enzyme digestion. This allows the easy assembly and ligation of any desired DNA sequence following the BioBricks standard. The concept is dependent on the specific restriction sites, which often appear in bacterial genomes, and, therefore, limits the insert length to the frequency of the restriction sites. The iBrick uses homing endonucleases (HEs, I-SceI and PI-PspI), enabling the cloning of big fragments since the HE recognition sites are longer (> 18 bp) and, therefore, rather rare in natural DNA sources. The iBrick strategy is suitable for assembling whole gene clusters in vectors using the BioBricks concept. As iBrick still has some limitations, such as low efficiencies and difficulties in capturing large BGCs from *Actinomyces*, iCatch was developed [89]. The target regions are released by genomic DNA (gDNA) digestions with HEs (as the target is flanked by I-SceI and PI-PspI sites) which are in vivo introduced via homologous recombination. The digestion takes place in an agarose gel plug to protect the DNA and the target fragments are cloned by self-ligation and transferred by electroporation into *Escherichia coli*. The iCatch is mainly an update on the precise “catching” of BGCs for further editing and assembly by applying the previously introduced iBrick method. Using this method, the actinorhodin (ACT) BGC from *S. coelicolor* was successfully obtained with an efficiency of 95.8% correct clones and heterologously expressed in a fast-growing thermophilic *Streptomyces sp.* strain. Furthermore, the obtained ACT cluster was further assembled into other iBrick components, which enabled their subsequent use for other studies [89].

#### 2.1.2. Direct Pathway Cloning (DiPaC)

Direct Pathway cloning (DiPaC) [92] accomplishes the assembly of complete biosynthetic pathways by covering full BGCs with long-amplicon polymerase chain reaction (PCR), and the introduction of homologous nucleotide overhangs allows subsequent in vitro HiFi DNA assembly such as Gibson assembly. Instead of HiFi DNA assembly, sequence- and ligation-independent cloning (SLIC) [93] can also be used which is not as time consuming, because the terminal homologous sequences of the fragments anneal in vitro and the stitching of the gaps takes place in vivo after transformation, inside the *E. coli* cell [94]. DiPaC is particularly suitable for cloning of short to midsized BGCs with low- and high-GC DNA. Not just fast cloning, but also refactoring of natural product pathways, is possible with DiPaC. Using the DiPaC method, the successful transfer of the erythromycin BGC from *Saccharopolyspora erythraea* into a *Streptomyces* heterologous host was reported [92].

#### 2.1.3. Artificial Gene Operon Assembly System (AGOS)

The artificial gene operon assembly system (AGOS) [95] is a “plug-and-play” method for the reconstruction and artificial assembly of gene operons of natural product pathways. The tool provides a set of entry plasmids (pAO-A → pAO-D, based on a pBluescript II SK (+) derivative), which are designed for the artificial gene operon manufacturing. Those plasmids contain an “entry cassette” which is comprised of two target sites, a controllable promoter (*tcp*_830_), a ribosome binding site (RBS) and specific restriction sites (XbaI and SpeI). Selection markers and specific intrinsic terminators are also incorporated, which delivers gene context-independency of each artificial gene operon. The target gene operon regions can be cloned into the entry plasmid by using the same restriction sites (XbaI and SpeI), which can either be done by de novo synthesis of the target region including the restriction sites, by introducing the sites during PCR amplification of the target regions, or via RED/ET-mediated recombination [96]. A derivative of the novBG01 cosmid [97], called mrsMR02, is the destination vector for the final assembly. It contains the genes needed for conjugational transfer and integration into the host genome as well as a multiple recombineering site for RED/ET-mediated recombineering [96]. The final assembly of the gene operons, present in the entry plasmids, takes place via the RED/ET-mediated recombination into the target sites on the destination vector. As proof of concept, Basitta et al. disassembled the well-studied 20 kb novobiocin BGC and successfully reorganized it again using AGOS [95].

#### 2.1.4. Modified Gibson Assembly for Cloning Large High CG DNA Fragments

Gibson Assembly is a well-known and commonly used method in synthetic biology [98]. It enables the one-pot, seamless assembly of multiple large DNA fragments (up to 900 kb) into a linearized vector of choice, only requiring overlapping sequences of neighboring DNA molecules. The reaction mixture includes a T5 exonuclease, which produces terminal single-stranded DNA overhangs on the provided fragments, which are subsequently repaired by a DNA polymerase and sealed by a DNA ligase. This classical Gibson reaction turned out to be rather ineffective for the cloning of large high GC content DNA fragments. The high GC overlap-ends on the linearized vector self-ligated with a rate of 80% [99]. To solve this problem, two universal terminal single-stranded overhangs with high AT contents are added to the ends of the vector. This does not only decrease the level of self-ligation to 45%, but it also makes the modified vector suitable for repeated use in future assemblies. Additionally, the introduction of two restriction enzyme sites (NdeI/NheI) into the respective side of the overhangs facilitates the hierarchical and seamless assembly of large DNA molecules. With those improvements, the assembly efficiency of the PII (pristinamycin II) BGC from *Streptomyces pristinaespiralis* HCCB10218 was increased from 2.5% (using the classical protocol) to 20%–40% (using the modified version) [99].

### 2.2. Introduction of Genetic Constructs into the Host: Delivery and Engineering Tools

The introduction of constructs for genetic manipulation of actinomycetes requires robust vehicles and methods for effective transfer and intracellular maintenance of the introduced material. The most commonly used vehicles for genetic engineering of actinomycetes are replicative and integrative vectors. Less frequently, “jumping genetic elements”, such as transposons, are applied [85]. The main difference between the replicative and integrative plasmids is the fact that replicative plasmids contain origins of replication and are able to multiply in the host cell (low-, mid and high copy number plasmids), while integrative plasmids harbor integrases and sequences which facilitate their integration into the actinomycete′s chromosome (integrative element-based plasmids, sequence homology-based plasmids). In contrast to integrative plasmids which integrate into the chromosome at certain position (via *att*-sites, actinomycetes integrative and conjugative elements (AICE) [100] or via homologous fragments flanking the target region), transposons can “jump” into the chromosome at different sites. Therefore, transposons were often exploited for “random” mutagenesis [101]. Replicative and *att*-site-based integrative plasmids are frequently applied for gene (over)expression, while plasmids containing homology fragments serve for homologous recombination and are mostly used for targeted gene inactivation [74].

In addition to the origin of replication and the integration function, the molecular vehicles contain other genetic parts that support the cloning, ensure the functionality of the constructs and the genetic engineering purpose as well as the selection and identification of potential mutant clones (Figure 2). Those genetic parts include the multiple cloning site (MCS), constitutive or inducible promoters, RBSs, transcriptional terminators, selection markers and/or reporter systems [85]. In the past, usually single genetic parts were introduced or exchanged in plasmids or other vehicles to build new constructions. Using synthetic biology this concept was recently advanced in order to combine several genetic parts (also called modules) at once (“plug-and-play”) (Section 2.2.2) [80].

Most of the molecular vehicles were originally applied for genetic manipulation of streptomycetes and over time expanded to other actinomycetes [52,102]. The commonly used replicative and integrative vectors, transposons as well as the standard methods for their introduction into actinomycetes (transformation, conjugation) were described in numerous original publications and recent reviews [74,85,103,104,105,106,107,108,109]. However, in the past five years (2015–2020) several new or modified genetic parts (e.g., promoters, RBS and other regulatory elements), engineered replicative as well as integrative systems, and CRISPR/Cas-based devices were added to the “genetic toolbox” for refactoring the genomes of *Actinomycetales* strains.

#### 2.2.1. Genetic Parts and Other Regulatory Elements for Engineering of Actinomycetes Genomes

To facilitate an efficient transcription/translation of sequences (e.g., gene (over)expression/protein production) in actinomycetes, diverse constitutive and inducible systems are applied. The most known and used constitutive systems are ermE*p [110], SF14*p [111], kasO*p [112]. Dynamic regulation of gene or pathway expression can be achieved by applying metabolite-responsive promoters such as the thiostrepton-(tipA*p) [113] or ε-caprolactam inducible promoter (nitA*p) [114], quorum sensing (operator/repressor) systems [115,116] (e.g., *tetO*–TetR [117], *gylR* and *gylP1/P2* [85,118]), pleiotropic/pathway-specific regulators (e.g., SARP family, TetR family) [119,120,121,122], and protein/RNA-based biosensors (e.g., TetR-based biosensors) [123,124,125,126,127,128]. Some of these concepts were initially developed in *E. coli* and/or *Saccharomyces cerevisiae* and later adapted to actinomycetes. For details, interested readers are referred to recently published reviews [72,74,81,129,130,131,132].

In following, we provide the reader with numerous examples of new and updated systems for engineering of actinomycetes.

##### Newly Identified, Synthetic and Modified Promoters and Other Genetic Regulatory Elements for Construction of Expression Plasmids

The use of diverse technologies including omics (RNA-seq, Ribo-seq and TSSseq), reporter assays and other methods resulted in the development of workflows for screening and/or designing of transcription- and translation-relevant sequences (e.g., promoter-, Shine-Dalgarno- (or RBS), terminator regions) [133]. These may have the potential to be applied in engineering of a wide range of actinomycetes. In addition, the already established systems (e.g., *Streptomyces lividans* strains containing glucuronidase (GusA)) were utilized for diverse modifications (e.g., variation of the distance between the Shine-Dalgarno (SD) domain and the start codon [134]) resulting in refactored as well as new synthetic promoters [135,136,137,138,139]. Their features and examples of successful applications for engineering of actinomycetes genomes were presented in several publications [72,74,129,131,134,140,141,142,143].

Recently, Luo et al. used the data obtained from RNA-seq analysis and identified 32 promoters in *Streptomyces albus* J1074. The promoters were cloned and further characterized in a *xylE*-based reporter assay and qPCRs [144]. The analysis of the enzymatic activity and transcription levels demonstrated that in the pool of 32 promoters, there are 10 strong and 4 constitutive promoters including housekeeping and heat/cold shock gene promoters. For the 10 strong promoters a 2 to 10-fold increase of gene expression levels was observed [144], when compared to the expression driven by the ermE*p. Furthermore, the authors selected five out of the 10 strong promoters and used them for activation of the polycyclic tetramate macrolactam (PTM) gene cluster from *Streptomyces griseus* in heterologous systems (*S. lividans* 66, *S. alb*us J1074, and *S. coelicolor* M1146). In contrast to the native strain *S. griseus*, heterologous hosts harboring the refactored gene cluster produced the PTM and the production yields were increased [144] compared to a previously described F60 construction [145]. These results suggest that the identified promoters may be widely applicable in synthetic biology platforms for activation of silent natural product biosynthetic pathways and characterization and/or optimization of already expressed BGCs in actinomycetes strains.

Similar study of genome wide screening and evaluation of promoters using transcriptome microarray data was conducted for *S. coelicolor* M145 [146]. A total of 8 out of 941 identified promoters were validated by a green fluorescent protein (GFP) reporter and real-time reverse-transcription quantitative polymerase chain reaction (RT-qPCR) in *S. coelicolor*, *Streptomyces venezuelae*, and *S. albus* and confirmed to drive a stable gene expression. Furthermore, four promoters were subjected to a “plug-and-play” platform to control the expression of the cryptic cluster of jadomycin B in *S. venezuelae* ISP5230. This led to different levels of the production of jadomycin B that corresponded to promoter strength [146]. Such microarray- and eGFP reporter assays-based approaches for identification of constitutive promoters were also successful for the industrial producer of natamycin *S. chattanoogensis* L10 [69,147]. The study resulted in the detection of the *thlM4*p promoter. The expression levels of genes under the *thlM4*p were sevenfold higher compared to the levels obtained with ermE*p. Application of the *thlM4*p promoter in an engineering experiment resulted in an increase of 30% of natamycin product yields compared to the same construction harboring ermE*p instead of *thlM4*p [147]. The same research group identified another strong promoter (*groES*p) in *S. chattanoogensis* L10 by using a proteomics-based approach [148].

The well-known GusA-reporter system was utilized for the same purpose of promoter identification in *Actinoplanes sp.* SE50/110, the natural producer of acarbose. RT-qPCR and mapping of the transcription starts allowed the identification of promoters with medium to strong expression [149].

An interesting approach was reported by Li et al. [150]. The authors used known inducible promoters for identification of the most optimal conditions for BGC expression in *S. coelicolor* (M145-OA) and *S. venezuelae* (Sv-Potr). Optimal induction conditions were determined by a response surface model, and, finally, inducible native promoters were identified by transcriptional analysis. Native promoters, which have shown a similar transcription profile to the inducible promoter under the optimal condition, were utilized to replace the inducible promoter following an elaborate replacement approach. With this strategy, it was possible to improve the titers of ACT and oxytetracycline (OTC).

The promoter kasO*p belongs to strong constitutive promoters. Bai et al. used the promoter region as a “template” and randomized the nucleotides downstream of the −10 sequences of kasO*p (library 1) as well as modified the spacer sequence between the −10 and −35 regions of kasO*p (library 2) [151]. Thereby, a set of 180 promoters was generated. Six (five from library 1 and one from library 2) synthetic promoters have shown a stronger activity than the original promoter (kasO*p). Furthermore, the activities of the synthetic promoters varied from 0.95% to 187.5%, and, therefore, the gradient strength of some of the promoters was tested in a proof-of-principle approach using the cryptic lycopene cluster. The functionality of the promoters could be confirmed in *Streptomyces*, as the lycopene BGC was activated and the compound was produced [151].

Zhao et al., used the heterologous σ70^hrdB^-dependent promoters, previously described as inefficient tools for gene expression in *Streptomyces*, and fused them with optimized 5′-untranslated regions (5′-UTRs) [152]. After modification of these systems (e.g., core promoter region of tac*p from *E. coli* was fused with the 5′-UTR_R15_ from *kasO*_R15_*p yielding tac*p), the σ70 -dependent promoters were efficiently recognized by *Streptomyces* housekeeping factor σ^hrdB^. In addition, the 5′-UTR_R15_ was optimized by randomizing the RBS and rational design of artificial RBSs gaining *tac*_RBS3_*p, of which activity was tested for squalene expression. The *tac*_RBS3_*p promoter have shown to be 2.1, 3.6 and 17.6 times more active than tac*p, *kasO*_R15_*p, and tac*p, respectively [152].

Ji et al. constructed a library of highly randomized, synthetic regulatory sequences, which varied in the sequence of the constitutive promoter as well as in the RBS region [137]. A NRPS responsible for the production of the blue pigment indigoidine was used as rapid screening assay of a large pool of the regulatory sequences. Based on the outcome of this study, the strength of the regulatory sequences was classified into the strong, medium and weak. For example, such a pool of synthetic regulatory sequences with different strengths is of advantage whenever a fine-tuning of the gene expression is required in order to achieve the maximal production yields of a compound. The utility of the synthetic regulatory sequences for promoter engineering of natural product BGCs has been shown for instance for the actinorhodin BGC [137].

In contrast to constitutive systems, inducible systems enable a controlled gene or pathway expression. Recently, such synthetic inducible regulatory systems were optimized for modulation of secondary metabolite production in *Streptomyces* [138]. The authors of the study employed the modular design concept [83] to build a high performance synthetic inducible regulatory system. The inducible regulatory system consisted of three separate functional genetic modules: (1) the induction module, (2) the target expression module and (3) the repressor expression module. Already known and well-characterized repressor-based induction modules for streptomycetes, including anhydrotetracycline (TET, TetR/*tetO*), oxytetracycline (OTR, OtrR/*otrO*), cumate (CMT, CymR/*cmtO*) and resorcinol (ROL, RolR/*rolO*) [117,153,154], were selected for this approach [138]. Each of these induction modules were combined with the SF14_RS_ repressor expression- and the kasO*_RS_ target expression module. In total, four inducible regulatory cassettes (IRCs) were designed and stepwise optimized by testing their performance in heterologous expression of the indigoidine NRPS and the actinorhodin type II PKS genes using *S. albus* J1074. The cumate-based synthetic inducible regulatory system has shown a large dynamic range (66.4× fold increase). Such inducible systems or their parts can be inserted into various plasmids to enable tight control of metabolite production in actinomycetes. This was for example shown for the PnitA-NitR [114] construction that resulted in a set of inducible *Streptomyces*-*E. coli* shuttle vectors [155].

##### Riboswitches for Biosensors

Another way to regulate gene expression is to use natural or modified riboswitches [156,157]. Riboswitches are non-protein coding RNAs that regulate diverse cellular processes, including transcription and translation. These regulatory parts at the 5′-region of the mRNA control the expression through allosteric alterations of the structure. The detailed regulation via riboswitches is described in other reviews [157,158]. As riboswitches are selective and specific to different ligands, they were exploited for the development of biosensors and became an important tool of synthetic biology.

In general, biosensors are composed of three units: (I) a signal input module (transcription factors (TFs) or riboswitches), (II) a regulatory module (TF-dependent promoters) (III) and a signal output module (reporter genes) [81,159]. In 2015, a protocol for application of the synthetic theophylline-dependent riboswitch [160,161] in *S. coelicolor* was published. Only 85 nt are need to be inserted between the start site of transcription and the start codon of the target gene to successfully control gene expression [156]. Additionally, in 2016 a novel modular dual control system was developed for coupled gene regulation at transcriptional and translational levels at the same time [162]. This “innovation” is based on the theophylline riboswitch [161] and the resorcinol and cumate-inducible system [153]. The system is applied to facilitate highly inducible control and complete silencing in the absence of both inputs. The dual control system is specifically designed for *Streptomyces* and *Actinobacteria* and resembles the first example of completely tight systems reported for *Actinobacteria* [162].

More recently, an antibiotic-specific whole-cell biosensor was developed for screening and optimization of antibiotic producers [128]. The system was derived from the TetR transcriptional repressor and successfully used to improve production of the polyketide antibiotic pamamycin. The optimization of the biosensor by alterations of the promoter and operator of output module and the ligand affinity of transcription factor module resulted in promising outcomes. Possibly, this and additional biosensors will be applied for generation of new actinomycetes cell factories to enable a more efficient manufacturing of bioactive compounds.

#### 2.2.2. Integrative and Replicative Expression Systems for Actinomycetes

Most of the integrative vectors which are used in genetic engineering of actinomycetes involve site-specific DNA recombination systems derived from phages (ΦC31 [163], ΦBT1 [164], VWB [165], TG1 [166], SV1 [167], R4 [168]) or from the integrative plasmid pSAM2 (λ-integrase) [169].

Vectors, which harbor the *ΦBT1* site, integrate at the unique *attB* site that is localized in the *SCO4848* gene (*S. coelicolor* genome) or into its orthologues in other streptomycetes [164]. The integration of a plasmid at this position into streptomycetes genomes obviously inactivates the gene *SCO4849* which is co-transcribed with *SCO4848*. This may affect the cell differentiation and result in delayed spore germination that can be complemented by expression of *SCO4849*. To avoid this polar effect, Gonzalez-Quiñonez et al. modified the ΦBT1 integrative vector pMS82 [164] by introducing a copy of *SCO4849* under the control of the promoter region of *SCO4848*. This resulted in the construction of the plasmids pNG1-4 (NG1 contains SCO4848*p + *SCO4849*, pNG2 contains SCO4848*p + *SCO4849* and ermE*p + RBS + MCS + *fd-ter,* pNG3 contains SCO4848*p + *SCO4849* and *bla* (resistance to ampicillin), and pNG4 contains SCO4848*p + *SCO4849*, ermE*p + RBS + MCS + *fd-ter* and *bla*). In two of these plasmids (pNG2 and pNG4), a copy of the ermE*p promoter was cloned for ensuring gene overexpression. As pNG3 and pNG4 contain the *bla* gene (ampicillin resistance), selection in *E. coli* is possible. The introduction of the system into mutants of *S. griseus*, *S. lividans* and *S. clavuligerus* carrying plasmid integration in *SCO4849* or in its orthologues resulted in a restored phenotype. The advantage of using pNG1-4 plasmids is that the integrative vectors do not affect the cell differentiation and lead to a neutral phenotype in streptomycetes. The approach can be transferred to other integrative vectors which cause similar effects. Another solution for avoiding such an undesired polar effect due to integrations of plasmids at certain positions into the genome of actinomycetes, might be the use of alternative integration sites. In 2017, Fogg et al. described a new *Streptomyces* phage, ΦJoe [170]. The actinophage encodes a serine integrase and belongs to the largest *Streptomyces* phage cluster (R4-like). The *int-attP* locus of ΦJoe was cloned into pSET152 [108] by removing the ΦC31 *int-attP* part and inserting the sequence of the newly identified system (*int-attP* locus of ΦJoe). This resulted in the final plasmid pCMF92. The activity of the ΦJoe system was demonstrated in in vitro recombination assays and in vivo, in *Streptomyces* (e.g., *S. venezuelae*) and *E. coli*. The plasmid pCMF92 is now available for use in *Streptomyces* species and might be expanded to other actinomycetes in the future.

Similar to pCMF92, the *E. coli*–*Streptomyces* shuttle vector pDYN6902 was derived from pSET152 [108,171,172]. The integrative expression vector contains the original thiostrepton-inducible tipA*p and the *E. coli* low copy number replication control of the F (fertility) factor. The copy-number of this vector is 12 times lower (one or two copies per cell) compared to that of pIJ6902, which is of advantage for cloning and expression of toxic genes. The functionality of pDYN6902 was confirmed in *Streptomyces ambofaciens* (ATCC23877 and DSM40697) and in *S. coelicolor* A3(2).

Since 2015, several vectors were developed using the concept of modular construct assembly (also called “plug-and-play” strategy). For example, SEVA (Standard European Vector Architecture) is a web-based resource [173] which was developed for easier construction of optimal plasmids based on a fixed architecture [174,175]. The SEVA database allows for the generation of a non-ambiguous nomenclature and indexes for a repository of functional sequences and final constructs. Originally, the system was established for the deployment of complex prokaryotic phenotypes in Gram-negative bacteria, such as *Escherichia* or *Pseudomonas*. The SEVA users are provided with a protocol to design the desired plasmid by choosing three functional cassettes named modules: origins of replication, antibiotic selection markers and a variety of cargoes with different applications. The backbones of the vector and the modules were minimized and edited to remove nonessential sequences and at the same time retain their functionality. Furthermore, the modules are flanked by uncommon restriction sites that enable the users to interchange the cassette within the respective module for the assembly of the most optimal construct. Very recently, the SEVA principle was utilized for cloning of 23 novel shuttle vectors to expand the genetic toolbox for *Streptomyces* and other actinomycetes [176]. Because the ORI module of this collection of integrative (*attP* integration system), low-copy number (SCP2* replication origin) and medium-to-high-copy number (pIJ101 replication origin) vectors contains an origin of replication for Gram-negative bacteria and streptomycetes, the plasmids can be used in both groups of organisms. In addition, the maker module was varied to gain vectors with different resistance markers (apramycin, chloramphenicol, kanamycin and/or streptomycin). The functionality of these constructions was confirmed in heterologous systems by successful expression of GFP and the production of the plant flavonoid apigenin in *S. albus* J1074 [176].

Another example of modularly assembled, standardized vectors which were developed for refactoring of BGCs in actinomycetes, such as *Streptomyces* species, are the systems reported by Aubry et al. [80]. A set of twelve constructs (pOSV801-pOSV812) provides combinations of different resistance cassettes and four orthogonal integration sites. The inserted FLP (flippase) recombination (site-specific recombination) [177] target sites are enabling the recycling of the antibiotic resistance marker and thus, they reduce the risk of undesired homologous recombination in *Streptomyces* strains whenever additional vectors are used. Due to the modular “architecture” and the presence of compatible part, the modules can be easily exchanged. The integrative plasmids (pOSV801-pOSV812) contain 5 variable modules. Module 1 contains the *E. coli* p15A origin of replication and one of the FRT (flippase recognition target) sites [177] for the Flp recombinase mediated excision. The second module (module 2) represents the antibiotic resistance marker (hygromycin-, apramycin- or kanamycin resistance gene). Module 3 contains the second FRT site as well as the origin of transfer. In Module 4 the integration cassette (ϕBT1, ϕC31, pSAM2, VWB) is located. Finally, the last module (module 5) is the cloning module which enables an iterative assembly of genes (or sequences) using the BioBrick approach (Section 2.1) [82]. The functionality of the 12 vectors was verified by their integration into the chromosome of *S. coelicolor* M145, *S. lividans* TK23, and *S. albus* J1074. For example, using pOSV802 and the BioBrick method, the albonoursin gene cluster from *Streptomyces noursei* was cloned and introduced into *S. coelicolor* M145 by intergeneric conjugation. The obtained mutant was confirmed to produce albonoursin, which demonstrated the applicability of the system for engineering of actinomycetes.

Taken together, the modular concept (e.g., SEVA) and the recently assembled shuttle vectors as well as the public availability of sequences for exchanging the modules within the vector are of advantage for semi high-throughput generation of plasmids for engineering of actinomycetes.

##### Multiplex Integration Systems for Site-Specific Genome Engineering

In addition to the classical integrative vectors which involve one type of attachment/integration site, a couple of systems for multiplex integration into the host´s chromosome were established.

The multiplexed site-specific genome engineering strategy (MSGE) [178] is based on the “one integrase-multiple *attB* sites” concept. It enables a stable integration of multiple copies of target BGCs into one chromosome in a single step. Therefore, additional artificial *attB* sites have to be introduced into the genome of the host strain. The introduction of the additional *attB* sites can be accomplished by using CRISPR/Cas9 editing tools (e.g., CRISPR/Cas9 described by Huang et al. [179]) (Section 2.2.4). Such a modified strain is then used to introduce multiple copies the target BGCs via conjugation, which leads to multiplex integration of the BGC into the native and artificial *attB* sites. Using this strategy, a *S. pristinaespiralis*-expression strain with five additional copies of the pristinamycin II (PII) BGC was created. The fermentation of this strain resulted in the highest titres reported to that date [178]. In 2019 an updated “plug-and-play” toolkit, specifically designed for actinomycetes, was published (advanced MSGE (aMSGE)) [180]. It enables high-efficient, multi-locus integration of BGCs into heterologous expression hosts. The tool consists of 27 synthetic plasmids with either a single or multiple integration sites (up to four). The toolkit facilitates the integration of BGCs, shortening it to a single step by allowing the ligation of several integration modules into plasmids through Gibson cloning and simultaneously insertion using different native *attB* sites of the strain. The aMSGE was successfully applied in *S. coelicolor* and *Streptomyces hygroscopicus* [180].

Similar systems for multiplex integration of genetic constructs were presented by Phelan et al., [181]. The authors constructed three standardized, orthogonal integration vectors (pAV (apramycinR, *attP*^VWB^), pSC (spectinomycinR, *attP*^ΦC31^) and pTB (thiostreptonR/beta lactamR, *attP*^ΦBT1^) as a basis for simultaneous integration at all three *attB* sites present in the genome. Each of the vectors harbors an origin of replication (*ori* pMB1), selection marker (apramycin-, spectinomycin- or thiostrepton resistance gene), RP4 origin of transfer, an *attP* site, a gene encoding an integrase (VWB, ΦC31 or ΦBT1), identical 5′- and 3′-UTRs flanking the promoter with a RBS and the target gene for expression as well as unique restriction sites. The promoters included the ermE*p, gapdh(EL)*p and rpsL(RO)*p native promoters which were recently identified from *S. albus* [144], and synthetic promoters. In total, 45 derivatives were cloned using the basic vectors pAV, pSC and pTB. These expression systems were exploited for the characterization of heterologous promoters and various *attB* chromosomal integration sites for protein expression in *S. venezuelae*. This approach resulted in the identification of 15 promoters that possess relative strengths that vary over three orders of magnitude [181]. Moreover, the authors observed differences in protein expression depending on the site of chromosomal integration of the expression plasmid.

A similar strategy of multiplex integration to this, proposed by Phelan et al. [181], was reported by Ko et al. [182]. The integration functions of the two closely related actinophages, ΦOZJ and ΦWTR [182] were investigated. It was shown that plasmids containing the ΦOZJ *attP*-int integrated into genomes of a pool of selected actinomycetes, including biotechnologically relevant strains [182]. Based on this outcome, a complementary set of plasmids with compatible *E.coli* replicons, resistance determinants and orthogonal ΦC31, ΦBT1 or ΦOZJ integrating functions was constructed. The resulting plasmids pJMD5, pJMD13 and pJMD14, respectively, were transferred simultaneously into *S. venezuelae*, *Streptomyces roseosporus* and *S. pristinaespiralis* in a single conjugation experiment [182].

As the tools for multiplex integration simplify the introduction of multiple genes or operons into a host´s genome and seem to facilitate a more predictable product biosynthesis, their application may contribute to less-time consuming engineering of a broad range of streptomycetes and possibly other actinomycetes.

#### 2.2.3. Transposon- and Homologous Recombination-Based Systems for Actinomycetes Engineering

In the past, transposons (e.g., Tn5) [183,184,185], homologous recombination [85,186,187,188,189,190] and site-specific recombination based on Cre/*loxP*, Dre/*rox* [101,191] as well as I-SceI [192,193] systems were extensively used to generate mutants in actinomycetes strains [41,101,194,195,196,197,198,199,200,201].

Transposon-based genome mutagenesis is mainly applied for systematic genetic studies of microorganisms [202,203]. However, the first *Streptomyces* transposons derived from Tn5, or IS493 were sub-optimal in terms of their transposition frequency and/or non-random mutations [85,129]. Later, a codon-optimized, hyperactive Tn5-based transposition system for in vivo random mutagenesis of *Streptomyces* genomes was developed [195,204,205]. It was demonstrated that the hyperactive transposase-based Tn5 transposition system integrates more randomly into the genome of *S. coelicolor*. This system was utilized to investigate the regulation of prodiginine in *S. coelicolor* [205].

The engineering of some of the non-*Streptomyces* species belonging to the order *Actinomycetales* (e.g., *Planobispora* sp., *Kibdelosporangium* sp., *Amycolatopsis* sp.) remains difficult as many of the established methods and tools fail in genetic manipulation of these strains (Figure 1). A new suicide vector p6SUI5ERPSL was constructed and optimized by Meyer et al. [206] to facilitate the deletion of genes in *Amycolatopsis* sp. The backbone of p6SUI5ERPSL includes an apramycin resistance gene (*aacC*), pBR origin of replication for *E. coli* (*oriV*) and the erythromycin promoter (ermE*p) upstream of the gene coding for 30S ribosomal subunit protein S12 from *Saccharopolyspora erythraea* (*rpsL*). RpsL confers sensitivity to streptomycin to clones which contain the p6SUI5ERPSL construct. This system can only be applied in hosts that are resistant to streptomycin due to mutations of the *rpsL* gene [207]. As *Amycolatopsis sp.* ATCC 39,116 is sensitive streptomycin, resistant mutants were generated, isolated and used to introduce constructs based on p6SUI5ERPSL, which contained flanking region for homologous recombination in *Amycolatopsis* ATCC 39116. With this strategy, three gene clusters (aldehyde oxidase (*yagRST*), vanillic acid decarboxylase (*vdcBCD*) and vanillate demethylase (*vanAB*)) were deleted in streptomycin resistant *Amycolatopsis* ATCC 39,116 derivatives. Therefore, the p6SUI5ERPSL system might serve as foundation for genetic modification of difficult to manipulate actinomycetes.

The I-SceI endonuclease [200] and the Cre recombinases [101,191] have been used to achieve large deletions in actinomycetes genomes.

I-SceI recognizes a rare 18-bp sequence (TAGGGATAACAGGGTAAT) and introduces double-strand breaks (DSBs) at this position. Such DSBs promote double-crossover recombination events. As double stranded breakage of the chromosome is lethal, only those cells of which genomes undergo homologous recombination can survive. So far, I-SceI recognition sites were not found in genomes of known and analyzed streptomycetes. Thus, I-SceI homing meganuclease and its recognition sequence can be exploited for genome editing in streptomycetes. Systems containing a codon-optimized I-SceI gene were developed for this purpose and successfully applied in *S. coelicolor* [200,208]. For instance, Fernández-Martínez and Bibb constructed the plasmid pIJ12738 plasmid which contained the recognition site for I-SceI and regions flanking the target sequence that was supposed to be deleted in the chromosome of *S. coelicolor* M1141. The plasmid was transferred into the strain, and the generated mutant clones were used as recipient for the delivery of the vectors pIJ12739 and pIJ12742, which harbor a codon optimized I-SceI meganuclease gene for expression in actinomycetes. Due to the activity of the I-SceI meganuclease and homologous recombination, the exconjugants revert to wild type or recombine their genomes to the engineered genotype. To demonstrate the functionality of the system, parts of the BGC of the undecylprodiginine complex of compounds were deleted in *Streptomyces coelicolor* M1141. Approximately half of the obtained clones possessed the desired marker-less genotype [208].

Recently also the Cre/*loxP* system was utilized for excision of a relatively large genomic region. In this case, 1.3-Mb and 0.7-Mb were deleted in the natamycin producer strain *Streptomyces chattanoogensis* L10 [69]. Two derivatives, *S. chattanoogensis* L320 and L321, were obtained and proposed for the developed of chassis for efficient polyketide production.

#### 2.2.4. CRISPR/Cas–Based Editing Tools for Actinomycetes

Since its identification as clustered regularly short palindromic repeats (CRISPR) in 2002 [209] and the discovery of its defense functions against phages [210], the system has been used as a precise genetic manipulation tool in a multitude of organisms [211,212,213,214,215]. The CRISPR defense is based on “cooperation” of CRISPR arrays and CRISPR associated (Cas) genes [216,217], which can be split into three major stages: the adaptation, expression and interference stage. The whole CRISPR/Cas mechanism has been reviewed in detail previously [218,219,220]. For the application as editing tool, only the interference stage is utilized. Required for target cleavage are only the Cas9 nuclease (in most cases derived from the type II CRISPR/Cas system from *Streptococcus pyogenes*), a CRISPR RNA (crRNA) and a trans-activating crRNA (tracrRNA). The crRNA is a short RNA, which is encoded as spacer in the CRISPR array and matches a previously encountered foreign nucleic acid. The tracrRNA is also a short RNA which helps with the processing of the crRNA and its recruitment to Cas9. The Cas9/crRNA/tracrRNA complex can introduce DSBs at any sequence that matches the crRNA and contains a recognition motif, called protospacer adjacent motif (PAM). When applied as editing tool, the gene regions for crRNA and tracrRNA can be fused together to build one single synthetic guide RNA (sgRNA) [221]. For the cleavage of any site of choice, only a sgRNA targeting the desired locus must be designed. The specific target cleavage can either be applied in vivo or in vitro. After target cleavage in in vivo systems, the DBS are either repaired by homology directed repair (HDR) (in cases in which a homologous template DNA is available) or by non-homologous end joining (NHEJ) (in cases in which no template is present). By simultaneous introduction of a homologous template DNA, precise genome modifications can be achieved. The biggest advantage of the CRISPR/Cas technology is the fact that basically any site of interest can be targeted without laborious requirements. However, disadvantages like off-target effects [222] and Cas9 toxicity [223,224,225] must be considered.

##### CRISPR/Cas9-Mediated Genome Editing Using pCRISPomyces

The pCRISPomyces was designed for precise *Streptomyces* engineering, presenting the possibility of multiplex genome editing by chromosomal deletions [223]. The tool includes a codon optimized *cas9* (from *S. pyogenes*) on a plasmid along with a tracrRNA and CRISPR array expression cassette in case of pCRISPomyces-1 or a sgRNA expression cassette for pCRISPomyces-2. Both versions of the plasmid contain several useful features such as a *B*bsI-flanked lacZ cassette for Golden Gate assembly of the spacer sequences, an XbaI site for addition of other elements such as editing templates for recombination-driven repair via Gibson assembly or traditional ligation, the colE1 origin for replication in *E. coli* and a RP4 origin for conjugational transfer as well as a temperature sensitive pSG5 *rep* region [226] for rapid clearance of the plasmid. Along with the target specific 15 bp protospacer sequence (3 bp PAM with 12 bp additional unique protospacer bases) a 2 kb editing template consisting of two homologous arms corresponding to the up- and downstream sequences of the target gene can be introduced into the plasmid. This feature prevents the tool from off-target effects and ensures precise deletion of the target sequence in the genome. Multiplex targeting is possible by addition of two different sgRNA cassettes. Using this method, an efficiency of 70%–100% for targeted chromosomal deletions between 20 bp and 30 kb in three different *Streptomyces* species was achieved. Furthermore, significantly higher editing efficiencies were observed using sgRNA targeting (pCRISPomyces-2) compared to tracrRNA/crRNA targeting (pCRISPomyces-1) [223]. There are several examples of application of the CRISPR/Cas9 tool in diverse strains, including *Actinoplanes* sp. [227] and a new *Streptomyces* species *Streptomyces formicae* [228].

##### CRISPR/Cas9-Mediated Genome Editing Using pKCcas9dO

pKCcas9dO is a tool for one-step CRISPR/Cas9-mediated genome editing. It was developed almost at the same time as the one published by Cobb et al., which was described above. Similar to the other toolkit, this high-efficiency editing system is also based on a pSG5 temperature sensitive plasmid harboring the thiostrepton-inducible promoter *tipA* and a codon optimized *cas9* from *S. pyogenes* along with two homology-directed repair templates corresponding to the flanking regions of the target sequence and a target specific sgRNA [179]. The system was used by Huang et al. to create single gene deletions as well as whole BGC deletions of up to 82.8 kb in *S. coelicolor* with an efficiency of 60%–100%. Multiplex BGC deletion was carried out with an efficiency of 45%–54% and even single point mutations could be introduced with an efficiency of 64%. This expands the CRISPR/Cas9-mediated genome editing abilities to deletion of nearly 83 kb-BGCs as well as the precise introduction of single point mutations. Another successful example for application of this CRISPR/Cas9 system is the MSGE-based engineering of *S. coelicolor* and *S. pristinaespiralis* [178] (Section 2.2.2).

##### CRISPR/Cas9-Mediated Genome Editing Using pCRISPR-Cas9

Almost simultaneous to the two tools mentioned before, a third CRISPR/Cas9 toolkit was delivered in 2015 for precise and efficient genetic manipulation. Similar to pKCcas9dO [179], the pCRISPR-Cas9 vector [229], that is presented here, contains the *tipA* promoter and a codon-optimized *cas9* accompanied by a designable sgRNA, which is inserted into NcoI and SnaBI restriction sites for easy exchange. An additional restriction site (StuI) is available for insertion of other elements such as homologous recombination templates. The vector is also based on a temperature-sensitive plasmid [230], which enables easy curing after the induction of Cas9 and the genetic engineering. This is another example of a CRISPR-Cas9 device that simplifies the genetic manipulation of high GC actinomycetes and provides an alternative to the time expensive introduction of single- and double cross over events in actinomycetes. The inactivation of two genes in *S. coelicolor* was possible without a homology-directed repair template, using the NHEJ pathway. However, low efficiencies ranging from 3% to 54%, depending on the used sgRNA, were observed. By optimization of the intrinsic NHEJ pathway via the co-expression of a DNA ligase, the generation of random-sized deletions around a precisely defined target position was achieved with an increased efficiency of 69%–77%. The addition of a homologous recombination template to the vector increased the deletion efficiency to nearly 100% [229]. Using this method, the sceliphrolactam BGC from a newly isolated *Streptomyces sp.* strain was identified [231]. Furthermore, the tool supported the elucidation of the dynemicin enediyne and anthraquinone pathway in *Micromonospora chersina* [232].

In addition, a system for reversible control of the gene expression of target genes was invented. This was named CRISPRi and stands for a CRISPR/dCas9-based interference system. As the name suggests, it is based on a catalytic dead version of Cas9 (dCas9) and uses a sgRNA targeting the promoter region. The dCas9 forms a roadblock on the DNA by binding to the promoter region without cutting the DNA, which can be easily reversed by inducing the loss of the pCRISPR-dCas9 plasmid [229].

##### CRISPR/dCas9-Mediated Multiplex Gene Repression

Further development on the CRISPR/dCas9 systems was performed by Zhao et al. to enable multiplex gene repression in the model strain *S. coelicolor* [233]. The integrative plasmid pSET152 with an encoded codon optimized *dCas9* and sgRNA, both under the control of constitutive promotors (*j23119* and ermE*p), was designed. After the integration of the plasmid into the genome, the dCas9 and sgRNA form a complex, bind the target site and stay there as roadblock unable to insert DSBs. This causes the repression of the gene expression. As the expression plasmid is integrated into the chromosome, this CRISPRi system is more stable compared to the temperature-sensitive pSG5-based system used before. The transformation efficiency with the pSET152-based plasmid backbone is also higher compared to pSG5-based plasmid transformations. By the insertion of up to four sgRNAs in the CRISPRi system, it ensures multiplex precise gene repression with the introduction of only one plasmid construct. This leads to a 2%–32% reduction of the target genes mRNA levels compared to the control. However, the repression efficiency of target genes becomes weaker when four genes are repressed at the same time compared to the repression efficiency of repressing just a single gene at a time. Using this method, functional gene screening in *S. coelicolor* was performed, which led to the identification of an orphan response regulator involved in bacterial growth [233].

##### CRISPR/Cas Base Editing System (CRISPR-BEST)

The CRISPR/Cas Base Editing SysTem (CRISPR-BEST) [234] comprises an adenosine (CRISPR-aBEST) and a cytidine (CRISPR-cBEST) deaminase-based base editor. CRISPR-aBEST can specifically convert an A:T base pair to a G:C base pair and the CRISPR-cBEST can convert a C:G base pair to a T:A within a target window of approximately 6–7 nucleotides. This technique is completely DBS-free and uses a Cas9 nickase (Cas9n) and sgRNA complex as delivery system for the deaminases to the target region. The base editing enables the introduction of stop-codons or loss-of-function mutations, without the formation of DSBs, which must be repaired in a certain way. Unbiased, genome-wide off-target evaluation showed a low level of off-target effects, which enables the application for broad genome editing studies. To expand the application of CRISPR-BEST, a Cys4-based sgRNA multiplexing system was also developed [234], which uses the type I-F Cas endoribonuclease Cas6 [235], also known as Cys4, instead of Cas9. Cys4 can self-process the sgRNA array and enables the expression of multiple sgRNAs with one single promotor and terminator, only separated by the Cys4-recognition sites. This is advantageous compared to approaches where each sgRNA needs an individual and independent transcription cassette for multiplex editing. CRISPR-BEST editing was successfully applied in *S. coelicolor*, *Streptomyces griseofuscus* and *S. collinus* [234]. Additionally, the CRISPy-web tool [236] was updated for identification and design of sgRNAs with regards to the application for CRISPR-BEST.

##### CRISPR/Cas9-CodA(sm) Combined System

In this approach, a CRISPR/Cas9 system is combined with a counter selective CodA(sm) [237] system. The D314A mutant of cytosine deaminase (CodA) converts supplemented 5′-flurocytosine (5FC) into 5′-flurouracil (5FU) which is highly toxic. This counter selective marker for *Streptomyces* and other bacteria was already reported in 2009 [237]. The delivery plasmid contains the codon-optimized *cas9* (from *S. pyogenes*), a target-specific sgRNA, *codA(sm)* and *aac(3)IV* resistance cassette for selection as well as homologous sequences to the flanking regions of the target sequence. The plasmid is introduced in the actinobacteria through conjugation. Next, DSBs are created by the sgRNA-Cas9 co-activity. The genome is then repaired via homologous recombination using the provided sequences on the plasmid. The mutant strains which have spontaneously lost the delivery plasmid can then be selected using the CodA(sm) system, resulting in unmarked mutant strains. This allows the reuse of the same delivery vector for successive gene replacements. When the system was applied in *S. coelicolor* for editing the actinorhodin polyketide chain length factor gene, 99% of the tested plasmid-free *S. coelicolor* exconjugants were correct mutants [238]. This method was also applied in *Streptomyces fradiae* for manipulation of the neomycin BGC [239].

##### CRISPR/Cas9 Knock-In Strategy

In this approach, the strategic knock-in of promoters is conducted to activate silent BGCs in *Streptomyces*. This one-step strategy uses the pCRISPomyces-2 plasmid [223] to strategically replace the native promoter regions of main biosynthetic operons or pathway-specific activators with strong, constitutive promoters (kasO*p) which work in several *Streptomyces* species. Using this strategy, the activation of three previously uncharacterized BGCs in *S. roseosporus*, *S. venezuelae* and *Streptomyces viridochromogenes* was possible [240].

##### Generic CRISPR/Cas9 Approach Using the Same sgRNA for Editing

Editing of multicopy genes and mobile genetic elements, which have highly similar and repetitive DNA sequences might be very challenging in actinomycetes. A generic two-step approach using CRISPR/Cas9 was designed to specifically target one copy of a multicopy gene of interest [241]. Therefore, a non-replicative plasmid, containing “bait DNA” and flanking homologous regions of the target gene is integrated into the genome via recombination. Subsequently, the “bait DNA” is targeted using a generic sgRNA, resulting in a DSB near to the target gene. The repair of the DSBs leads to either reversion or gene copy-specific editing. Using this strategy, the deletion of the native copies of senogeneic silencers *lsr2* paralogs in *S. ambofaciens* was achieved [241].

##### Fine-Tuning of Cas9 Expression

One of the biggest drawbacks while working with CRISPR/Cas9 genome editing tools is the toxicity of Cas9 expression for the cells [223,224,225]. The level of Cas9 expression, therefore, must be tightly controlled. When reducing the Cas9 level in the cell, transformation efficiency can be increased but it can also lead to insufficient Cas9 activity. To find the perfect balance, which is also dependent on the used expression strain, a plasmid toolkit was created. This allows for controlling the Cas9 expression during CRISR-Cas9-mediated recombination experiments. The expression is either translationally regulated using theophylline or under the control of a relatively weak constitutive promoter [225].

##### CRISPR/Cas9 TAR Cloning Approach

TAR (Transformation associated recombination)-Cloning [242] allows for isolation of large chromosomal regions without the effort of constructing a random clone library. The method is based on in vivo homologous recombination of a specific genome target and a linear TAR-Cloning vector containing anchor-like, specific terminal targeting sequences that are homolog to the sequence of interest. The method includes a co-transformation, in which the vector and genomic DNA are introduced into yeast followed by the homologous recombination of the flanking regions of the target sequences and the vector anchors. This results in circular Yeast Artificial Chromosomes (YAC) containing the target sequence. Using the standard TAR protocol, it is possible to clone DNA fragments up to 250 kb but the efficiency of obtaining clones with the correct construct is 1%–5%, which is very low. The efficiency can be increased to 30% by introducing DSBs into the chromosomal DNA near (>100 bp) the target region, typically performed by restriction enzymes. This leads to more efficient homologous recombination between the cloning vector anchor and the target genomic sequences, as they are embedded in shorter DNA fragments. In a newer approach the specificity of TAR cloning was increased by using CRISPR/Cas9 to introduce the DSBs [243]. This offers the advantage that nearly any sequence of interest can be cloned into a YAC vector, without the need of specific and rare restriction enzymes, which cut near the target sequence but not within. This method was applied for cloning parts of the pristinamycin BGC from *S. pristinaespiralis* [244] and/or the prodigiosin BGC from *S. coelicolor* [245].

##### Cas9-Assisted Targeting of Chromosome Segments (CATCH)

The Cas9-assisted targeting of chromosome segments (CATCH) [246] is a direct cloning approach, which uses Cas9-directed cleavage to excise the target fragments for cloning. Cell lysis and in vitro cleavage of the chromosomal DNA by sgRNA-guided Cas9 takes place in agarose gel plugs to protect the DNA from shearing. The digested DNA is then purified from the gel plugs and ligated into the bacterial artificial chromosome-vector (BAC) of choice by Gibson assembly. This allows one-step cloning of near-arbitrary, long bacterial genomic sequences up to 100 kb. Positive-rates of 90% were achieved when cloning the jadomycin gene cluster (36 kb) from *S. venezuelae* or the chlortetracycline gene cluster (32 kb) from *Streptomyces aureofaciens*. However, with the increasing fragment size, the efficiency decreased, showing only a 21% positive-rate when cloning 100 kb sequences [246].

##### Gibson Assembly Combined with CRISPR/Cas9

The classical Gibson assembly requires a linearized vector backbone into which the target fragments are ligated. Commonly, the vector is linearized either by restriction enzyme digestion or inverse PCR [98]. In this combined approach, the vector is cleaved using CRISPR/Cas9 and a specific designed sgRNA. It is a quick and convenient way to use any desired vector without the need of suitable restriction sites or appropriate length for PCR amplification. The target fragments are then assembled into the CRISPR/Cas9 digested vector following the standard Gibson protocol [247]. This was successfully applied for refactoring BGCs from *S. pristinaespiralis* and *S. coelicolor* [178].

##### In vitro Packaging Mediated One-Step Targeted Cloning

This approach combines the specific nicking, mediated by CRISPR/Cas9 and in vitro λ packaging for targeted cloning of natural product pathways [248]. The BGC of interest is released from the genome by in vitro cleavage with a target-specific sgRNA:CRISPR/Cas9 complex and subsequently ligated with the EcoRV-linearized pJTU2554 vector. Both the insert and vector have blunt-ends, which should lead to mostly correct ligation-products, as the remaining non-target gDNA is most likely to have none. Furthermore, the blunt-end ligation approach allows universal use of the EcoRV-linearized vector, as no overlapping regions to the target sequence are needed. The ligation mixture is in vitro packaged into λ phage particles, which are then used to infect *E. coli* cells. The cloning efficiency ranges from 18%–54%, depending on the insert size (preferably between 37.4 and 50.4 kb). This approach was successfully used to clone the pathways of Tü3010 (*stu*) from *Streptomyces thiolactonus* NRRL 15,439 and sisomicin (*sis*) from the genetically intractable *Micromonospora inyoensis* DSM 46,123 strain [248].

In the past five years (2015–2020) several CRISPR/Cas9-based tools were established and successfully applied to engineer streptomycetes strains and some species of other genera within *Actinomycetales* (Table 2). It is likely that those molecular devices will be further optimized and become even more “popular” than the traditional systems for genetic manipulation of actinomycetes.

## 3. Summary and Conclusions

Molecular tools are essential for the genetic engineering of organisms, including actinomycetes-the important producers of bioactive compounds. In general, genetic engineering workflows involve the design and assembly of constructs, the introduction of these constructs into the host cell and intracellular processes that result in genetically modified strains and usually new phenotypes.

In the past, several methods and tools were developed to facilitate these steps. In most cases, established vehicles such as vectors (integrative or replicative systems) are used as backbone of the construction. These systems often already contain important genetic parts (origin of replication and/or integration functions, genes encoding CRISPR/Cas systems, selection markers and/or reporter systems, MCS, constitutive or inducible promoters, RBSs, transcriptional terminators). However, additional parts can be introduced into the construct. These parts can be amplified in a PCR reaction or excised from already existing sources. In case of actinomycetes DNA, the amplification and assembly of several fragments (e.g., cloning of BGCs) might be challenging because of the high GC-content of the DNA. This problem can be solved by using recently improved polymerases and optimized buffers in combination with fast assembly methods. For example, it was demonstrated that the implementation of iCatch, DiPaC, AGOS and modified Gibson Assembly resulted in functional constructs for actinomycete engineering. Instead of using the available genetic parts that might be inefficient in some actinomycetes, new or modified elements can be utilized. Recently, several engineering studies were published in which additional new or modified genetic parts (e.g., promoter-, RBS-engineering, components of biosensors) were identified and successfully utilized for fine-tuning and improvement of the production of actinomycete compounds.

After the desired genetic parts were assembled, the construct needs to be transferred into the actinomycete´s cell. This is usually accomplished using conjugation or protoplast transformation. Depending on which functions are provided on the construct, integration and/or replication take place. For instance, replicative and integrative plasmids are often applied for sequence/gene expression. Plasmids containing homologous fragments upstream and downstream of a target region are frequently used to facilitate the single- and double crossover for sequence/gene inactivation, including deletion. In particular, the traditional method for the generation of deletion mutants is very time-consuming. Recently, the revolutionary CRISPR/Cas system was adapted to actinomycetes for fast genome editing (e.g., insertion and expression, inactivation, deletion and repression of gene expression). In the meantime, several derivatives were reported (e.g., pCRISPomyces, pKCcas9dO, pCRISPR-Cas9, CRISPR/dCas9, CRISPR-BEST, CRISPR/Cas9-CodA(sm), pCRISPomyces-2, CATCH). Indeed, the implementation of the CRISPR/Cas systems resulted in the efficient genome engineering of several *Streptomyces* strains and some other actinomycetes.

In the past five years (2015–2020), terrific progress has been achieved in the field of molecular tools for genetic manipulation of actinomycetes. The newly developed tools provide new opportunities for natural product discovery and global metabolic engineering of actinomycetes. It is very likely that this progress will continue, and additional cell factories will be developed for manufacturing of various valuable products.

## Figures and Tables

**Table 1:** Advantages and disadvantages of cloning strategies and example of successful application.

Method	Advantages	Disadvantages	Example Organisms
**iCatch** [89]	+ cloning of large fragments + complements other cloning methods (like TAR and CATCH)	− previous strain editing (insertion of HE recognition sites) required	*S. coelicolor* [89]
**Direct Pathway Cloning (DiPaC)** [92]	+ refactoring of BGCs is possible + cloning of full BGCs	− PCR amplification of target sequence: risk of mutations − pure and high molecular weight gDNA required	*Saccharopolyspora erythreae* [92]
**Artificial gene operon assembly system (AGOS)** [95]	+ refactoring of BGCs is possible	− limited production of refactored BGCs	*Streptomyces niveus* [95]
**Modified Gibson Assembly for cloning large high GC DNA fragments** [99]	+ reusable vector + increased assembly efficiency for high GC content DNA	− PCR amplification of target sequence: risk of mutations	*S. pristinaespiralis* [99]

**Table 2:** Advantages and disadvantages of CRISPR/Cas-based genome editing and examples of successful application in actinomycetes.

Method	Advantages	Disadvantages	Example Organisms
**CRISPR/Cas9 engineering using pCRISPomyces** [223]	+ targeting any site of interest + multiplex targeting possible	− problems in *S. coelicolor* [249] − time-consuming elimination of temperature sensitive plasmid − Cas9 toxicity possible − Cas9 off-target effects possible	*S. viridochromogenes* [223] *S. albus* [223] *S. lividans* [223] *Actinoplanes sp.* [227] *S. formicae* [228]
**CRISPR/Cas9 engineering using pKCas9dO** [179]	+ targeting any site of interest + multiplex targeting possible + inducible Cas9 expression	− time-consuming elimination of temperature sensitive plasmid − Cas9 off-target effects possible	*S. coelicolor* [178,179] *S. pristinaespiralis* [178]
**CRISPR/Cas9 engineering using pCRISPR-Cas9** [229]	+ targeting any site of interest + inducible Cas9 expression	− time-consuming elimination of temperature sensitive plasmid − *tipA* in host genome required − Cas9 off-target effects possible	*S. coelicolor* [229] *Streptomyces sp. SD85* [231] *Micromonospora chersina* [232]
**CRISPR/dCas9-mediated multiplex gene repression** [233]	+ targeting any site of interest + stable system through integrative plasmid -multiplex approach	− Cas9 off-target effects possible − Cas9 toxicity possible − repression levels are lower when targeting multiple sites	*S. coelicolor* [233]
**CRISPR-BEST** [234]	+ targeting any site of interest + rescue approach when dCas9 was unsuccessful	− *tipA* in host genome required - Cas9 off-target effects possible	*S. coelicolor* [234]*S. griseofuscus* [234]*S. collinus* [234]
**CRISPR/cas9-CodA(sm)** [238]	+ targeting any site of interest + unmarked mutants + reusable delivery vector + no off-target effects	− Cas9 toxicity possible − Cas9 off-target effects possible	*S. coelicolor* [238] *S. fradiae* [239]
**CRISPR/Cas9 knock-in strategy** [240]	+ targeting any site of interest + activation of silent gene clusters	− requires introduction of recombinant DNA for activation of BGC, may be challenging for some strains	*S. albus* [240] *S. lividans* [240] *Streptomyces rhodeosporus* [240] *S. venezuelae* [240] *S. viridochromogenes* [240]
**Generic CRISPR/Cas9 approach** [241]	+ no specific sgRNA design required + limited off-target effects	− previous strain editing required	*S. ambofaciens* [241]
**Fine-tuning Cas9 expression** [225]	+ reduced Cas9 toxicity	− non-toxic expression levels must be explored for each strain	*S. coelicolor* [225] *S. lividans* [225]
**CRISPR/Cas9 TAR cloning approach** [243]	+ targeting any site of interest + less-time intensive compared to traditional TAR cloning + no advanced experience in working with yeast required	− working with yeast − Cas9 off-target effects	*S. pristinaespiralis* [244] *S. coelicolor* [245]
**Cas9-assisted targeting of chromosome segments (CATCH)** [246]	+ targeting any site of interest + one-step approach + cloning of large fragments + in gel cleavage protects gDNA from shearing	− size limit of 150 kb − Cas9 off-target effects possible	*S. venezuelae* [246] *S. aureofaciens* [246]
**Gibson assembly and CRISPR/Cas9** [247]	+ targeting any site of interest + no specific vectors or inverse PCR are required	− Cas9 off-target effects possible	*S. pristinaespiralis* [178] *S. coelicolor* [178]
**In vitro packaging mediated one-step targeting cloning** [248]	+ refactoring possible + no strict requirements for gDNA preparation	− fragment size limited to packaging capacity of 37.4–50.4 kb from λ phage	*S. thiolactonus* [248] *Micromonospora inyoensis* [248]

## Abbreviations

ACT	actinorhodin
AGOS	artificial gene operon assembly system
AICE	actinomycetes integrative and conjugative elements
aMSGE	advanced multiplexed site-specific genome engineering
BAC	bacterial artificial chromosome
BGC	biosynthetic gene cluster
CATCH	Cas9-assisted targeting of chromosome segments
CRISPR/Cas	clustered regularly interspaced short palindromic repeats/CRISPR associated
crRNA	CRISPR RNA
DiPaC	Direct pathway cloning
DSB	double-strand break
DNA	deoxyribonucleic acid
gDNA	genomic DNA
GFP	green fluorescent protein
GusA	glucuronidase
HDR	homology directed repair
HE	homing endonuclease
MCS	multiple cloning site
mRNA	messenger RNA
MSGE	multiplexed site-specific genome engineering
NHEJ	non-homologous end joining
NRPS	nonribosomal peptide synthetase
OTC	oxytetracycline
PCR	polymerase chain reaction
PKS	polyketide synthase
PTM	polycyclic tetramate macrolactam
qPCR	quantitative PCR
RBS	ribosome binding sites
Real-time RT-PCR	real-time reverse-transcription quantitative polymerase
RNA	ribonucleic acid
SD	Shine-Dalgarno
SEVA	standard European vector architecture
sgRNA	synthetic guide RNA
SLIC	sequence- and ligation-independent cloning
TAR	transformation associated recombination
tracrRNA	trans-activating CRISPR RNA
UTR	untranslated region
YAC	yeast artificial chromosome
